# Fibroblast-secreted hepatocyte growth factor mediates epidermal growth factor receptor tyrosine kinase inhibitor resistance in triple-negative breast cancers through paracrine activation of Met

**DOI:** 10.1186/bcr3224

**Published:** 2012-07-12

**Authors:** Kelly L Mueller, Julie M Madden, Gina L Zoratti, Charlotte Kuperwasser, Karin List, Julie L Boerner

**Affiliations:** 1 Department of Oncology, Wayne State University, 4100 John R. Street, Detroit, MI 48201, USA; 2 Department of Pharmacology, Wayne State University, 540 East Canfield Street, Detroit, MI 48201, USA; 3 Department of Anatomy and Cellular Biology, Tufts University School of Medicine, 136 Harrison Avenue, Boston, MA 02111, USA

## Abstract

**Introduction:**

Epidermal growth factor receptor (EGFR) tyrosine kinase inhibitors (TKIs) have shown clinical efficacy in lung, colon, and pancreatic cancers. In lung cancer, resistance to EGFR TKIs correlates with amplification of the hepatocyte growth factor (HGF) receptor tyrosine kinase Met. Breast cancers do not respond to EGFR TKIs, even though EGFR is overexpressed. This intrinsic resistance to EGFR TKIs in breast cancer does not correlate with Met amplification. In several tissue monoculture models of human breast cancer, Met, although expressed, is not phosphorylated, suggesting a requirement for a paracrine-produced ligand. In fact, HGF, the ligand for Met, is not expressed in epithelial cells but is secreted by fibroblasts in the tumor stroma. We have identified a number of breast cancer cell lines that are sensitive to EGFR TKIs. This sensitivity is in conflict with the observed clinical resistance to EGFR TKIs in breast cancers. Here we demonstrate that fibroblast secretion of HGF activates Met and leads to EGFR/Met crosstalk and resistance to EGFR TKIs in triple-negative breast cancer (TNBC).

**Methods:**

The SUM102 and SUM149 TNBC cell lines were used in this study. Recombinant HGF as well as conditioned media from fibroblasts expressing HGF were used as sources for Met activation. Furthermore, we co-cultured HGF-secreting fibroblasts with Met-expressing cancer cells to mimic the paracrine HGF/Met pathway, which is active in the tumor microenvironment. Cell growth, survival, and transformation were measured by cell counting, clonogenic and MTS assays, and soft agar colony formation, respectively. Student's *t *test was used for all statistical analysis.

**Results:**

Here we demonstrate that treatment of breast cancer cells sensitive to EGFR TKIs with recombinant HGF confers a resistance to EGFR TKIs. Interestingly, knocking down EGFR abrogated HGF-mediated cell survival, suggesting a crosstalk between EGFR and Met. HGF is secreted as a single-chain pro-form, which has to be proteolytically cleaved in order to activate Met. To determine whether the proteases required to activate pro-HGF were present in the breast cancer cells, we utilized a fibroblast cell line expressing pro-HGF (RMF-HGF). Addition of pro-HGF-secreting conditioned fibroblast media to TNBC cells as well as co-culturing of TNBC cells with RMF-HGF fibroblasts resulted in robust phosphorylation of Met and stimulated proliferation in the presence of an EGFR TKI.

**Conclusions:**

Taken together, these data suggest a role for Met in clinical resistance to EGFR TKIs in breast cancer through EGFR/Met crosstalk mediated by tumor-stromal interactions.

## Introduction

The tyrosine kinase receptor, epidermal growth factor receptor (EGFR), is a molecule overexpressed in triple-negative breast cancer (TNBC); that is, estrogen receptor-negative, progesterone receptor-negative, and HER2-negative. In fact, expression of EGFR is one of the defining characteristics of TNBC and is a predictor of poor prognosis [1]. Clinical testing of EGFR tyrosine kinase inhibitors (TKIs) in breast cancer patients led to the conclusion that EGFR TKIs are ineffective in treating this disease [2,3]. However, EGFR TKIs are in clinical use in lung, colon, and pancreatic cancers [4-6].

As with many targeted therapeutics, acquired resistance to EGFR TKIs is of growing concern in lung cancer. One molecule shown to contribute to the acquired resistance to EGFR TKIs is the tyrosine kinase receptor Met.

Met is a proto-oncogene that encodes the hepatocyte growth factor (HGF) receptor. HGF is the only known ligand of the Met receptor. Met amplification has been associated with acquired EGFR TKI resistance in lung cancer cell lines and human lung tumors containing EGFR tyrosine kinase domain mutations [4,7,8]. Resistance to EGFR TKIs in lung cancers and glioblastomas was overcome by inhibition of Met activity [9,10]. Met phosphorylation has also been identified as a contributor to EGFR TKI resistance in breast cancer [11]. Similar to the lung cancer models, sensitivity to EGFR TKIs was increased by co-treating these cells with Met TKIs [11]. However, in contrast to the lung cancer models, breast cancers are not initially sensitive to EGFR TKIs and therefore do not develop an acquired resistance in response to Met upregulation. Breast cancers appear to be intrinsically resistant to EGFR TKIs and therefore may regulate Met via a distinct mechanism.

Met has been shown to be phosphorylated prominently in TNBCs. However, Met is not commonly found to be amplified or mutated in these tumors [12,13]. Mechanisms of Met activation include both ligand-dependent and ligand-independent pathways. Classical activation and subsequent tyrosine phosphorylation of Met involves the processing and activation of pro-HGF by proteases after binding to the extracellular domain of Met [14,15]. Christensen and colleagues summarized a number of ligand-independent methods of Met phosphorylation in their review, which includes the following: mutation of Met, constitutive dimerization of Met associated with overexpression, pathway activation via hypoxic conditions, transactivation by other membrane proteins (including EGFR), and loss of negative regulators [16]. Many of these mechanisms are thought to be critical for the contribution of Met to tumorigenesis.

Here we provide evidence that production of HGF by neighboring stromal cells is a mechanism for EGFR TKI resistance in TNBCs. We found that TNBC cell lines without constitutive activation of Met were sensitive to EGFR TKIs in culture. Adding exogenous HGF to these breast cancer cell lines decreased sensitivity to EGFR TKIs. Knocking down EGFR expression decreased viability in TNBC cell lines. In contrast to HGF protecting cells from loss of cell viability upon inhibition of EGFR kinase activity, HGF added to cells with knocked down EGFR expression failed to show a recovery in viability. This observation suggests that EGFR/Met crosstalk is critical for mediating EGFR TKI resistance through an EGFR kinase-independent mechanism. In order to mimic EGFR/Met crosstalk in the tumor microenvironment we used conditioned media from HGF-producing fibroblasts as well as co-cultures of TNBCs with these fibroblasts, and again demonstrated that the activation of Met mediated EGFR TKI resistance. Taken together, these data demonstrate that activation of Met in TNBC cells can stimulate EGFR/Met crosstalk and subsequent EGFR TKI resistance.

## Materials and methods

### Cell lines, growth conditions, and reagents

The SUM149 breast cancer cell line was derived from an invasive ductal mammary carcinoma and the SUM102 breast cancer cell line was derived from an intraductal carcinoma with micro-invasion [17]. These cell lines were obtained from our colleague Dr Stephen Ethier (Medical University of South Carolina, Charleston, SC, USA). Reduction mammoplasty fibroblasts expressing human HGF (RMF-HGF) cells were generated by our co-author Dr Charlotte Kuperwasser (Tufts University, Boston, MA, USA) [18,19]. SUM149 cells were grown in 5% IH media (Ham's F-12 media supplemented with 5% fetal bovine serum, 1 μg/ml hydrocortisone, and 5 μg/ml insulin). SUM102 cells were grown in SFIHE media (Ham's F-12 media supplemented with 1 μg/ml hydrocortisone, 5 μg/ml insulin, 10 ng/ml epidermal growth factor, 5 mM ethanolamine, 10 mM HEPES, 5 μg/ml transferrin, 10 nM triiodo-thyronine, 50 μM sodium selenite, and 5% BSA). RMF-HGF cells were grown in DMEM + 10% fetal bovine serum media (DMEM media supplemented with 10% fetal bovine serum). All media were supplemented with 2.5 μg/ml amphotericin B and 25 μg/ml gentamicin sulfate.

The EGFR TKI gefitinib (Iressa) was provided by AstraZeneca (Wilmington, DE, USA). Erlotinib was purchased from LC Laboratories (Woburn, MA, USA). All other reagents were purchased from Thermo Fisher (Waltham, MA, USA) or Sigma (St Louis, MO, USA), unless indicated.

### Small hairpin RNA knockdown

To downregulate EGFR expression we used small hairpin RNA (shRNA) lentiviral particles using commercially available lentiviral constructs from Open Biosystems (Huntsville, AL, USA). Twenty-four EGFR shRNA constructs were screened and validated for EGFR knockdown. Four constructs targeting different nonoverlapping sequences of the EGFR mRNA were used in the studies. Specifically, the EGFR sequences targeted by the shRNAs are as follows: shRNA #1 targets CCACCAAATTAGCCTGGACAA (3,136 base pairs from ATG), shRNA #2 targets CCGTGGCTTGCATTGATAGAA (3,485 base pairs from ATG), shRNA #3 targets CAGCATGTCAAGATCACAGAT (2,544 base pairs from ATG), and shRNA #4 targets CCTCCAGAGGATGTTCAATAA (149 base pairs from ATG).

The lentiviruses were packaged using a third-generation lentiviral packaging system developed by Didier Trono and colleagues (Lausanne, Switzerland) and were purchased from Addgene (Cambridge, MA, USA) [20]. Specifically, Addgene plasmids pMLDg/pRRE (12251), pRSV-Rev (12253), and pMD2.G (12259) were transfected into HEK293T cells with the lentiviral vectors containing the shRNAs using FUGENE6 (Roche, Madison, WI, USA). Cellular supernatant was collected on days 2 and 3 after transfection, pooled, and filtered. The lentivirus was titered using HEK293T cells incubated with increasing concentrations of virus with polybrene and selected via the puromycin selection on the lentiviral vector. Colonies were counted and used to compare viral preps and between viruses for consistent titers used in experiments. Equal amounts of virus were added to cells in the presence of polybrene for 4 days prior to cell lysis or cell viability testing.

### Plasmid constructs

Kinase-dead EGFR was cloned into pcDNA3 as previously described [21]. Mutations were made within the sequence of EGFR encoded by shRNA #4 to abrogate shRNA binding to the re-expressed EGFR. Specifically, shRNA #4 recognizes CCTCCAGAGGATGTTCAATAA. We used site-directed mutagenesis to change the sequence to TCTCCAAAGGATGTTTAACAA and will refer to this construct as KD-EGFR^mt^.

### Isolation of conditioned medium

RMF-HGF fibroblasts were cultured to confluence, switched to serum-free DMEM and cultured for 24 to 48 hours in low-volume conditions (5 ml media/100-mm dish). Conditioned media were pooled from 24-hour and 48-hour serum-free incubations and HGF was quantified using a HGF-specific ELISA assay according to the manufacturer's instructions (R&D Systems, Minneapolis, MN, USA). Conditioned media were frozen in 1 ml aliquots and stored at -20°C.

### Co-cultures

For the immunoblotting studies, SUM102 or SUM149 cells were plated with RMF-HGF fibroblasts at the indicated cell number ratios for 48 hours in the normal growth medium of SUM102 or SUM149 cells. Lysates were prepared and immunoblotted with pMet and β-actin antibodies. For the BrdU incorporation assays, SUM102 or SUM149 cells were stained with CellTracker Green (Life Technologies, Grand Island, NJ, USA) and RMF-HGF cells were stained with CellTracker Orange. DNA synthesis was then assessed using BrdU incorporation for 4 hours after 24 hours of gefitinib treatment.

### Cell viability assays

For the MTS assays, cells were plated at 4,000 cells/well of a 96-well plate in triplicate from parental or EGFR knockdown cells. For comparison purposes, the parental cells were treated with 0.5 μM gefitinib with or without 50 ng/ml HGF as indicated. The knockdown cells were treated with or without 50 ng/ml HGF for 72 hours. The MTS reagent was added per manufacture directions (Promega, Madison, WI, USA) and was read using a Dynex spectrophotometer. The experiment was repeated three times, with error bars representing the standard error of the mean.

### Cell growth assays

For the proliferation assays the indicated breast cancer cells were plated in triplicate in six-well plates at 35,000 cells per well (day 0). The next day, and every other day thereafter for 7 days, the cells were treated with gefitinib at the indicated dosage. The number of cells was determined using a Coulter Counter (Beckman Coulter, Indianapolis IN, USA) on days 1, 4, and 8. Each experiment was repeated at least twice and the graphs represent the average and standard error of the mean at day 8.

### BrdU-incorporation assays

After co-culture with the fibroblasts for 24 hours, 0.5 μM gefitinib was added for an additional 20 hours. Then 100 μM BrdU was added for 4 hours prior to fixation of the cells with 4% paraformaldehyde for 20 minutes at room temperature. The cells were then permeabilized using 0.01% Triton X-100 for 3 minutes at 4°C and washed with PBS three times. DNA was then exposed by incubation of the cells with 2 M HCl for 1 hour at 37°C and neutralized with two borate buffer washes. The cells were blocked in 20% goat serum for 1 hour at room temperature and incubated with Alexa-Fluor 594 anti-BrdU conjugated antibody (1:50; Life Technologies) for 1 hour at 37°C. Excess antibody was washed away and the coverslips were mounted, and BrdU-incorporated nuclei were counted as a ratio of the total number of cells. One hundred cells were counted per coverslip, with each experiment performed in duplicate.

### Clonogenic survival assays

Cells were cultured for 7 days with the indicated concentration(s) of gefitinib in the presence or absence of 50 ng/ml active HGF or ~110 μM pro-HGF in conditioned media. Cells were trypsinized and plated at 2,000 cells/35-mm dish (without treatment) for 7 days. Colonies were stained with crystal violet and counted.

### Soft agar colony formation

Cells were plated in a 0.45% agar noble layer at 250,000 cells/well of a six-well plate. Gefitinib in the presence or absence of HGF or of HGF-containing conditioned media was added every other day for 3 weeks. Colonies were counted using Colony Counter software (Gel Count, Oxford, United Kingdom) and were averaged.

### Immunoblotting

Cells were plated at 1 million cells per 100-mm dish and cultured for 48 hours. Cells were then lysed in CHAPs lysis buffer (10 mM CHAPs, 50 mM Tris, pH 8.0, 150 mM NaCl, and 2 mM ethylenediamine tetraacetic acid with 10 μM NaOVa and 1× protease TKI cocktail; EMD Biosciences, Billerica, MA, USA). The indicated amount of protein lysate was separated by SDS-PAGE and transferred to Immobolin-P. Membranes were blocked in either 5% nonfat dry milk or 5% BSA for 1 hour at room temperature. The following primary antibodies were used in the experiments: anti-EGFR (1:500; Cell Signaling, Danvers, MA, USA), anti-Met (1:500; Cell Signaling), anti-pMet (1:500; Cell Signaling), and anti-β-actin (1:10,000; Sigma). The membranes were incubated with antibodies overnight at 4°C. The membranes were then washed with Tris-buffered saline + 0.1% Tween-2 three times for 10 minutes each, followed by incubation with the appropriate secondary antibody, and then with another series of three washes. Incubation with enhanced chemiluminescence (GE Biosciences, Piscataway, NJ, USA) followed by exposure to film was used to detect the reactive bands. Each experiment was repeated at least three times and quantitated using densitometry.

## Results

### HGF protects TNBC cells from a gefitinib-induced decrease in cell growth, survival, and transformation

**Figure 1:**
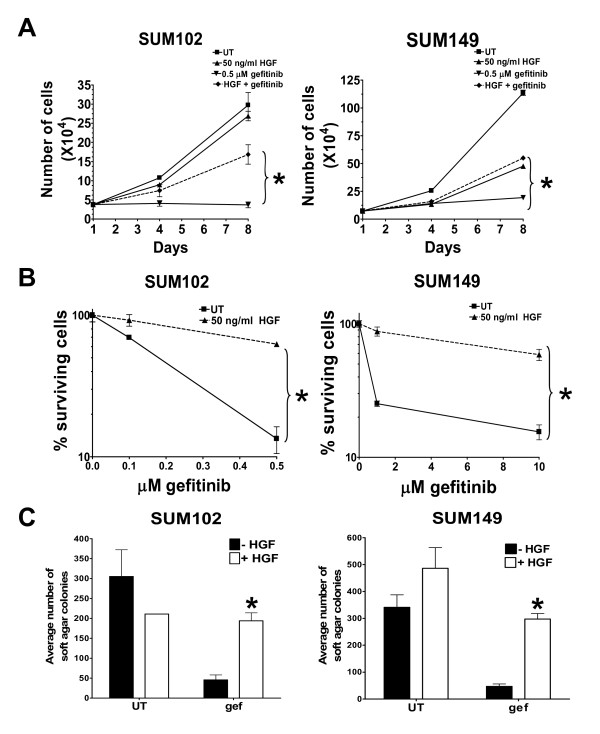
**Hepatocyte growth factor protects triple-negative breast cancer cells from gefitinib treatments**. **(A) **SUM102 and SUM149 cells were plated at 35,000 cells/well of a six-well plate on day 0. Under normal growth conditions, 50 ng/ml hepatocyte growth factor (HGF) and/or 0.5 μM gefitinib was added to the cells on day 1 and subsequently every other day. Cells were counted on days 1, 4, and 8 using a Coulter Counter. **(B) **SUM102 and SUM149 cells under normal growth conditions were treated with increasing concentrations of gefitinib in the presence or absence of 50 ng/ml HGF for 7 days. Cells were trypsinized and replated at 4,000 cells/35-mm dish and grown under normal growth conditions for 7 days. Colonies were stained with crystal violet and counted using a cell counter. The percentage of surviving cells was calculated by dividing the number of colonies on the treated plate by the number of colonies on the untreated plate. **(C) **Under normal growth conditions, SUM102 and SUM149 cells were plated in a layer of agar sandwiched between two layers of agar of different percentage. Every other day for 3 weeks, 50 ng/ml HGF and/or 0.5 μM gefitinib were added to the top layer in a final volume of 300 μl. Colonies were stained with *p*-iodonitrol and counted using a cell counter. Each experiment was performed in triplicate at least three times. Student's *t *test was used to determine significance between gefitinib-treated and gefitinib + HGF-treated cells. *P < 0.05. UT, untreated.

In the clinic, breast cancers do not respond to EGFR TKIs; however, a number of EGFR-expressing TNBC cell lines show sensitivity to EGFR TKIs [3,22] (Figure 1A). Met has been shown to crosstalk with EGFR to mediate EGFR TKI resistance both in cell culture models and in patients [7,23-25]. The ligand for Met, HGF is not expressed in epithelial cells; and Met is not phosphorylated in gefitinib-sensitive breast cancer cell lines [26]. To determine whether activated Met could promote resistance to EGFR TKIs, two EGFR TKI-sensitive TNBC cell lines (SUM102 and SUM149) were incubated with the EGFR TKI gefitinib in the presence or absence of 50 ng/ml HGF and the cell growth, survival, and transformation were measured.

SUM102 and SUM149 cells treated with gefitinib stopped proliferating by day 4 of treatment (Figure 1A, diamonds). However, when recombinant HGF was present (starting at day 1), SUM102 and SUM149 cells continued to proliferate in the presence of gefitinib (Figure 1A, dotted lines). These data suggest that activation of Met signaling by HGF is sufficient to stimulate cell growth when EGFR kinase activity is reduced. Similarly, HGF increased the clonogenic survival of SUM102 and SUM149 cells in the presence of gefitinib from 10% to 80% (Figure 1B). Lastly, anchorage-independent growth, as measured by soft agar colony formation, was increased four-fold to sixfold over the control after HGF treatment in the presence of gefitinib (Figure 1C). Taken together, these data suggest that Met activation provides an EGFR kinase-independent mechanism of cell growth, survival, and transformation.

### HGF stimulation of cell viability requires EGFR expression

**Figure 2:**
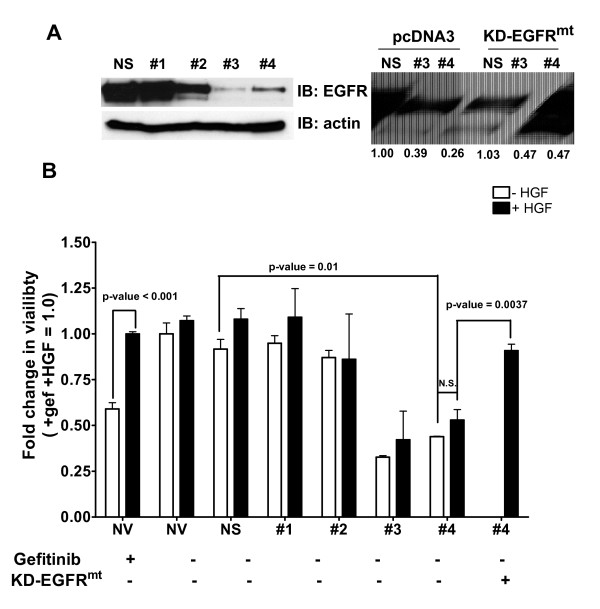
**Hepatocyte growth factor-mediated cell survival requires epidermal growth factor receptor expression**. SUM102 cells were transduced with lentivirus expressing a non-silencing control or four nonoverlapping epidermal growth factor receptor (EGFR) small hairpin RNA (shRNA) targeting sequences. In addition, SUM102 cells were treated with gefitinib (gef) or hepatocyte growth factor (HGF), or were transfected with pcDNA3 or KD-EGFR^mt ^as indicated. **(A) **Four days after transduction and/or transfection, cells were lysed, and lysates were used to determine expression levels of EGFR via immunoblotting. β-actin was used as a loading control. **(B) **Duplicate cells were trypsinized and replated in a 96-well plate. One series of gefitinib-treated cells were used as a control to demonstrate HGF protection from gefitinib treatment (far-left bar). Remaining cells were treated with or without HGF at 50 ng/ml without gefitinib for 72 hours. In addition, cells were transfected with KD-EGFR^mt ^at the same time as transduction with shRNA (far-right bar). Cell viability was measured using MTS assays. Numbers under the immunoblot indicate relative densitometry. Lines indicate comparison samples. Each experiment was performed in triplicate at least three independent times. *Gef ± HGF, *P *< 0.001; -HGF ± EGFR knockdown, *P *= 0.001. N.S., not significant; NV, no virus; NS, nonsilencing. #1, #2, #3, and #4, arbitrary shRNA clone numbers. KD-EGFR, kinase-dead EGFR; EGFR*, mutated sequence within the shRNA targeting region.

To determine whether HGF was simply signaling through Met independent of EGFR to increase cell viability, we knocked down EGFR expression and stimulated SUM102 cells with HGF. EGFR was knocked down using shRNA lentivirus. Four shRNA constructs targeting different nonoverlapping regions of the EGFR mRNA were used along with a nonsilencing control. Two of the shRNA constructs knocked down EGFR expression more than 90% of the nonsilencing control (Figure 2A, shRNAs #3 and #4). MTS assays were used to assess the viability in the presence of the shRNA constructs with or without 50 ng/ml HGF. The no-virus and nonsilencing controls as well as EGFR shRNAs #1 and #2 had no effect on cell viability, correlating with little to no decrease in EGFR protein expression (Figure 2B, white bars). Interestingly, knocking down EGFR with EGFR shRNAs #3 and #4 significantly decreased cell viability (Figure 2B, white bars). These results are similar to the results we published previously [22].

As shown through the growth, survival, and anchorage-independent growth assays in Figure 1, HGF increases cell viability in the presence of gefitinib in the SUM102 cells (Figure 2B, gefitinib). Adding HGF to the no-virus and nonsilencing controls as well as EGFR shRNAs #1 and #2 did not significantly change the viability as compared with that of untreated cells (Figure 2B, white vs. black bars). Importantly, when EGFR was successfully knocked down with EGFR shRNAs #3 and #4, HGF addition did not significantly increase cell viability (Figure 2B, white vs. black bars). In addition, re-expression of a kinase-dead EGFR, mutated to be unrecognized by shRNA #4, was able in part to recover the viability of SUM102 cells treated with HGF (Figure 2A, B). These data suggest that HGF/Met mediated viability and that EGFR TKI resistance requires EGFR expression but not EGFR kinase activity, indicating a critical role for EGFR/Met crosstalk.

### Conditioned media from HGF-expressing fibroblasts stimulates clonogenic survival in the presence of gefitinib

HGF is not expressed by epithelial cells and therefore Met requires HGF to be produced by surrounding stromal cells for ligand-dependent activation [26]. In the context of breast cancer, fibroblasts are a major component of the stroma and have been shown to express HGF [26]. Single-chain pro-HGF secreted by fibroblasts binds to Met with high affinity, but it is not signaling competent unless converted to active two-chain HGF by endoproteolytic cleavage [15,27]. Therefore, to determine whether TNBC cells sensitive to EGFR TKIs have the proper protease(s) to mediate cleavage of pro-HGF, activation of Met, and subsequent EGFR/Met crosstalk, we utilized conditioned media from a human fibroblast cell line engineered to express human HGF (RMF-HGF) [18,19]. We confirmed that HGF was present in its inactive single-chain form by western blotting in agreement with previous published reports (data not shown and [28]).

**Figure 3:**
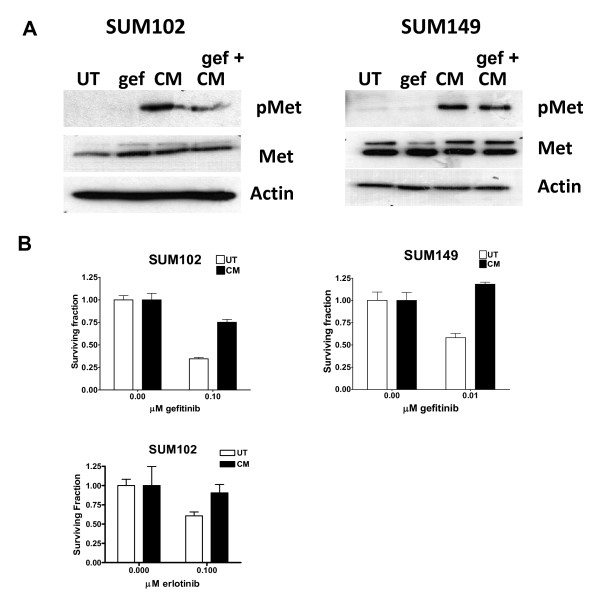
**Hepatocyte growth factor stimulates Met phosphorylation and enhances clonogenic survival in the presence of gefitinib**. **(A) **SUM102 and SUM149 cells were cultured with condition media from RMF-HGF fibroblasts for 1 hour in the presence or absence of 0.5 μM gefitinib (gef). Lysates were prepared and analyzed for Met phosphorylation using immunoblotting. Met and β-actin expression levels were used as controls. CM, pro-hepatocyte growth factor (pro-HGF) conditioned media; UT, untreated. **(B) **SUM102 and SUM149 cells were treated with conditioned media from RMF-HGF fibroblasts in the presence or absence of the indicated concentration of gefitinib or erlotinib for 7 days, adding fresh drug and conditioned media (CM) every other day. Cells were trypsinized, replated, and grown under normal growth conditions for 7 days. Colonies were stained using crystal violet and counted using a cell counter. Each experiment was performed in triplicate at least three times. Student's *t *test was used to determine survival differences between gefitinib-treated cells in the presence or absence of conditioned media.

The amount of pro-HGF in the conditioned media from the RMF-HGF was quantitated using an ELISA assay and ~100 nM pro-HGF was used for the experiment. Pro-HGF-conditioned media stimulated phosphorylation of Met in both the SUM102 and SUM149 cell lines (Figure 3A, CM). This phosphorylation of Met was not changed by co-treatment with the EGFR TKI gefitinib (Figure 3A, gef + CM). Clonogenic cell survival assays were performed with co-treatment of SUM102 and SUM149 with 0.5 μM gefitinib and/or 100 nM HGF conditioned media. Similar to treating the cells with recombinant HGF, pro-HGF stimulated clonogenic survival in the presence of gefitinib in both SUM102 and SUM149 cells (Figure 3B). To verify the significance of these findings, SUM102 cells were treated with another EGFR TKI, erlotinib, at 0.5 μM with 100 nM HGF-conditioned media and analyzed in a similar clonogenic assay. As seen with gefitinib, HGF-conditioned media protect SUM102 cells from erlotinib. These data suggest that SUM102 and SUM149 cells contain the one or more proteases capable of activating pro-HGF to active HGF, which induces Met phosphorylation and mediates subsequent EGFR/Met crosstalk.

### Co-cultures of HGF-expressing fibroblasts and TNBCs stimulate Met phosphorylation and DNA synthesis in the presence of gefitinib

**Figure 4:**
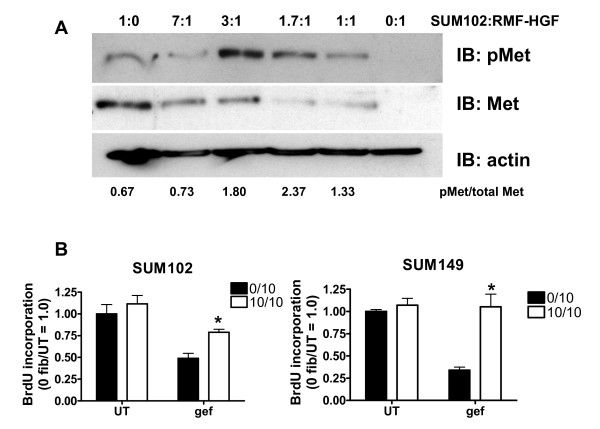
**Met phosphorylation and DNA synthesis in triple-negative breast cancer cells in the presence of gefitinib**. Co-culture with hepatocyte growth factor (HGF)-expressing fibroblasts increases Met phosphorylation and DNA synthesis in triple-negative breast cancer cells in the presence of gefitinib. **(A) **SUM102 cells were labeled with CytoTracker green and were co-cultured with RMF-HGF cells at different ratios. Lysates were analyzed for phosphorylation of Met by immunoblotting. β-actin and total Met protein expression were used as controls. Numbers below the immunoblots represent relative densitometry measurements of the ratio of pMet to total Met. **(B) **RMF-HGF fibroblasts were plated on coverslips. SUM102 and SUM149 cells were labeled with CytoTracker green and plated on top of the RMF-HGF fibroblasts at the indicated ratios. Then 1.0 μM gefitinib (gef) was added to the cells for 24 hours. SUM102 cells were incubated with BrdU for 18 hours and SUM149 cells were incubated with BrdU for 4 hours. Cells were fixed and stained using anti-BrdU-Alexa-Fluor-595. SUM102 and SUM149 cells labeled green were counted as BrdU-positive or BrdU-negative. Student's *t *test was used to determine statistical significance between gefitinib-treated cells with or without co-cultures. *SUM102, *P *= 0.0471; SUM149, *P *= 0.0028. UT, untreated; 0/10, fibroblast to tumor cell ratio; 10/10, fibroblast to tumor cell ratio.

To mimic the tumor microenvironment we performed co-culture experiments with RMF-HGF fibroblasts and SUM102 or SUM149 cells. SUM102 cells were co-cultured with RMF-HGF fibroblasts at various ratios and the phosphorylation of Met was determined. Using semi-quantitative densitometry to compare phosphorylated Met with total Met, co-culturing SUM102 cells with RMF-HGF fibroblasts increased Met phosphorylation with an increasing number of fibroblasts plated (Figure 4A, numbers below the immunoblot). RMF-HGF fibroblasts do not express Met and therefore are unable to stimulate Met phosphorylation in an autocrine fashion (Figure 4A, last lane). To determine whether the co-culture of RMF-HGF fibroblasts with SUM102 or SUM149 cells would rescue gefitinib-induced cell growth inhibition, BrdU incorporation after gefitinib treatment was quantitated (Figure 4B). BrdU incorporation was counted only in the SUM102 or SUM149 cells as identified by CellTracker green staining. As expected, BrdU incorporation was significantly reduced with gefitinib treatment (Figure 4B, gef). When the breast cancer cells were co-cultured with RMF-HGF fibroblasts, however, they were significantly less sensitive to gefitinib (Figure 4B, gef, white vs. black bars). These data provide, for the first time, evidence of a mechanism for EGFR TKI resistance in breast cancer: EGFR/Met crosstalk through interaction between stromal fibroblasts and cancer cells.

## Discussion

We have herein described a mechanism for EGFR TKI resistance in TNBC cells through activation of Met by its ligand HGF. Specifically, we found that HGF increased growth, clonogenic survival, and anchorage-independent growth in the presence of the EGFR TKI gefitinib. This enhanced growth in the presence of HGF and gefitinib was dependent on EGFR expression because knocking down EGFR expression decreased survival in the presence of gefitinib independent of HGF treatment. In addition, conditioned media from fibroblasts producing HGF as well as co-cultures with these fibroblasts stimulated Met phosphorylation and survival in the presence of gefitinib. Taken together, these results describe a role for stromal-produced HGF in intrinsic resistance to EGFR TKIs in TNBCs.

Crosstalk between EGFR and Met has been reported in breast, lung, and brain cancers [29]. This crosstalk has been suggested to occur via EGFR phosphorylation of Met as well as Met phosphorylation of EGFR. Phosphorylation of EGFR by Met has been shown to occur via direct as well as indirect mechanisms. With respect to direct phosphorylation, several groups, including our own, have demonstrated that Met associates with EGFR and that this association mediates transphosphorylation of EGFR [23,30]. Indirect methods of Met phosphorylation of EGFR include Met-dependent upregulation of EGFR ligands [31-33] and Met-dependent activation of other tyrosine kinases (for example, c-Src) [11]. The functional significance of this crosstalk has been reported by several groups. Engelman and colleagues demonstrated that crosstalk between EGFR and Met was mediated by phosphorylation and signaling from HER3 to Akt in lung cancer cell lines and that this crosstalk mediated resistance to EGFR TKIs [7]. We have previously shown that inhibiting Met kinase activity in breast cancer cell lines with constitutive Met activation sensitizes these cells to EGFR TKIs [11]. Here we expand our knowledge about Met/EGFR crosstalk by demonstrating not only that inhibition of Met increases sensitivity to EGFR TKIs, but also that activation of Met by HGF indeed promotes resistance to EGFR TKIs in TNBC cell lines. These data are supported by the work of others in which lung cancer cell lines containing activating mutations of EGFR that mediate sensitivity to EGFR TKIs can be made resistant in the presence of HGF [24]. In addition, Zhang and colleagues used a novel mouse model to demonstrate that HGF expression can promote tumor growth of EGFR-expressing breast cancers [25,34]. We have now demonstrated a role for HGF in conferring EGFR TKI resistance to TNBC cells.

In breast cancer, Met is thought to be activated through association with co-receptors or by binding to its ligand HGF produced by the tumor-associated stromal cells. Hochgrafe and colleagues found that Met is phosphorylated in breast tumors and that the phosphorylation of EGFR and Met is enriched in TNBC tumors [35]. EGFR and Met are expressed at high levels in these tumors and are both independent characteristic markers for TNBCs [36,37]. Expression of HGF, the ligand for Met, is limited to cells of mesenchymal origin, suggesting that communication between the tumor and the stroma is required for Met activation [26]. Here we have shown that HGF produced by human fibroblasts mediates Met phosphorylation and subsequent resistance to EGFR TKIs in TNBC cell lines. In addition, we have demonstrated in a co-culture system that HGF produced by neighboring fibroblasts promotes resistance to EGFR TKIs in SUM102 and SUM149 cells. We have thus provided evidence for the crosstalk between EGFR and Met to be mediated through interactions between the tumor cell and the microenvironment in TNBCs.

## Conclusions

TNBC is a subtype of breast cancer that overlaps with the basal-like breast cancers lacking estrogen receptor/progesterone receptor and HER2 expression. While the survival rates of women with estrogen receptor-positive and HER2-positive breast cancers have increased with the development of tamoxifen and herceptin, respectively [38,39], TNBCs retain the lowest 5-year survival rates [1]. The EGFR is overexpressed in 54% of TNBCs, yet EGFR TKIs remain ineffective for their treatment [2,3]. Our data here support the hypothesis that this lack of efficacy in clinical treatment of breast cancers with EGFR TKIs is due to crosstalk between EGFR and Met. In combination with our previous data and those of others, these results suggest that targeting EGFR and Met in combination in TNBC may be an effective therapeutic strategy.

## Abbreviations

BSA: bovine serum albumin; DMEM: Dulbecco's modified Eagle's medium; EGFR: epidermal growth factor receptor; ELISA: enzyme-linked immunosorbent assay; HGF: hepatocyte growth factor; PBS: phosphate-buffered saline; RMF-HGF: reduction mammoplasty fibroblasts expressing human hepatocyte growth factor; shRNA: small hairpin RNA; TKI: tyrosine kinase inhibitor; TNBC: triple-negative breast cancer.

## Competing interests

The authors declare that they have no competing interests.

## Authors' contributions

KLM performed the experiments producing the results shown in Figure 3. JMM performed the experiments producing the results shown in Figure 4. GLZ assisted with the experiments producing the results shown in Figures 3 and 4. CK provided the RMF-HGF fibroblasts for the study. KL helped conceive the ideas of the manuscript, helped with the design of the experiments, and assisted in editing the manuscript. JLB performed the experiments producing the results shown in Figures 1 and 2, conceived the ideas, designed the experiments, and wrote the manuscript. JLB also provided the funding for the manuscript. All authors read and approved this manuscript for publication.
